# Exome sequencing generates high quality data in non-target regions

**DOI:** 10.1186/1471-2164-13-194

**Published:** 2012-05-20

**Authors:** Yan Guo, Jirong Long, Jing He, Chung-I Li, Qiuyin Cai, Xiao-Ou Shu, Wei Zheng, Chun Li

**Affiliations:** 1 Center for Quantitative Sciences, Vanderbilt Ingram Cancer Center, Nashville, TN, USA; 2 Vanderbilt Epidemiology Center, Vanderbilt University, Nashville, TN, USA; 3 Department of Biostatistics, Vanderbilt University, Nashville, TN, USA; 4 Center for Human Genetics Research, Vanderbilt University, Nashville, TN, USA

**Keywords:** Exome sequencing, SNP, Target region, Capture efficiency

## Abstract

**Background:**

Exome sequencing using next-generation sequencing technologies is a cost efficient approach to selectively sequencing coding regions of human genome for detection of disease variants. A significant amount of DNA fragments from the capture process fall outside target regions, and sequence data for positions outside target regions have been mostly ignored after alignment.

**Result:**

We performed whole exome sequencing on 22 subjects using Agilent SureSelect capture reagent and 6 subjects using Illumina TrueSeq capture reagent. We also downloaded sequencing data for 6 subjects from the 1000 Genomes Project Pilot 3 study. Using these data, we examined the quality of SNPs detected outside target regions by computing consistency rate with genotypes obtained from SNP chips or the Hapmap database, transition-transversion (Ti/Tv) ratio, and percentage of SNPs inside dbSNP. For all three platforms, we obtained high-quality SNPs outside target regions, and some far from target regions. In our Agilent SureSelect data, we obtained 84,049 high-quality SNPs outside target regions compared to 65,231 SNPs inside target regions (a 129% increase). For our Illumina TrueSeq data, we obtained 222,171 high-quality SNPs outside target regions compared to 95,818 SNPs inside target regions (a 232% increase). For the data from the 1000 Genomes Project, we obtained 7,139 high-quality SNPs outside target regions compared to 1,548 SNPs inside target regions (a 461% increase).

**Conclusions:**

These results demonstrate that a significant amount of high quality genotypes outside target regions can be obtained from exome sequencing data. These data should not be ignored in genetic epidemiology studies.

## Background

Next-generation sequencing technologies have substantially decreased the cost of sequencing large genomic regions. It is still financially prohibitive, however, to perform whole genome sequencing for a large number of subjects, especially for large scale genetic epidemiology association studies, at a sufficient depth for accurate genotype calls. The exome represents about 1% of the human genome with approximately 30 million base pairs [1] but accounts for over 85% of all mutations identified in Mendelian disorders [2]. As a result, exome sequencing is currently an attractive and practical approach for investigation of coding variations.

**Figure 1:**
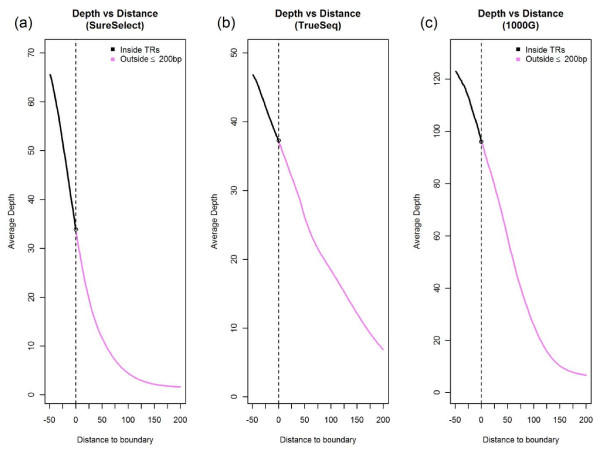
**Average depth around boundaries of target regions (1-50 bp inside and 1-200 bp outside boundaries).** Negative distance means inside a target region, and positive distance means outside a target region.

**Figure 2:**
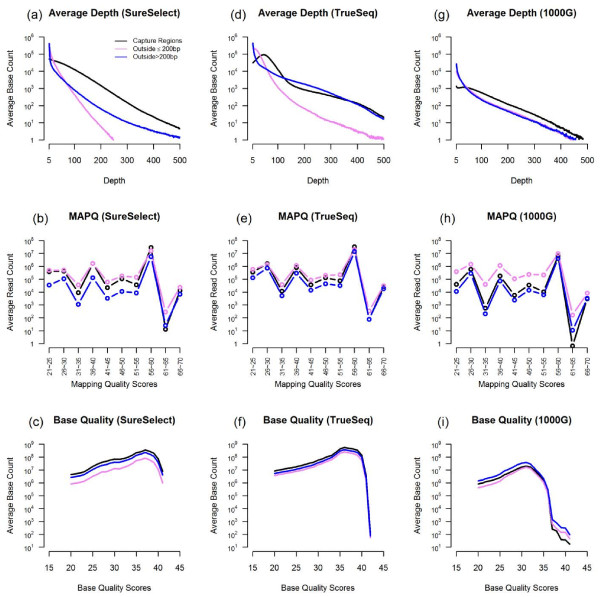
**Distributions of depth, mapping quality score, and base quality score for “Inside TR”, “Outside ≤200 bp”, and “Outside > 200 bp”**.

**Figure 3:**
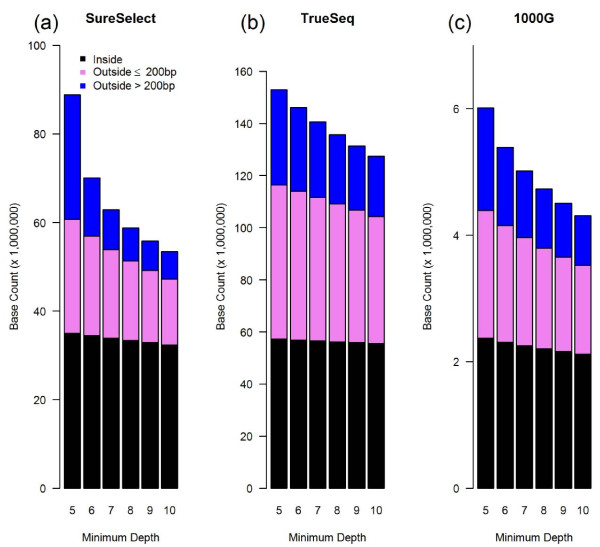
Distribution of sites with a minimum depth of 5 to 10.

**Figure 4:**
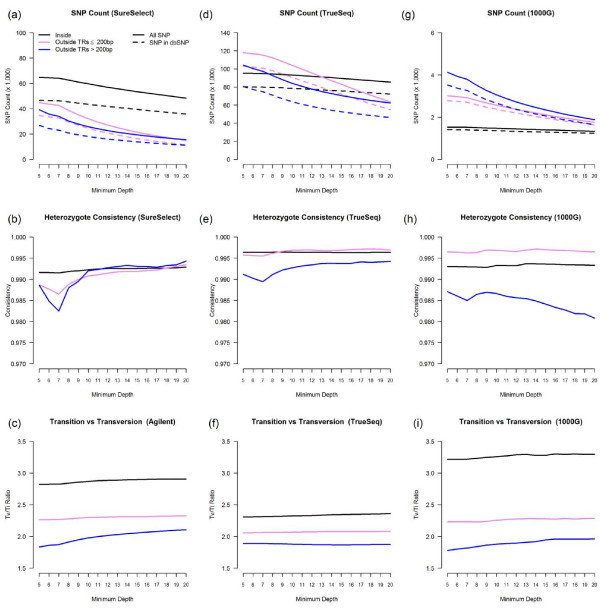
Average SNP count per sample, heterozygote consistency, and Ti/Tv ratio.

While exome sequencing primarily targets exons, noncoding regions such as introns, intron-exon boundary regions, UTRs, and intergenic regions can also be sequenced as a byproduct. It is well known that SNPs located in promoter and UTR regions may regulate gene expression. It was traditionally believed that introns were not as important as exons, but many studies have now established some functional significance for introns. For example, Rearick et al. [3] suggested that some introns can encode specific proteins and can be processed after splicing to form noncoding RNA molecules. Yi et al. [4] found a pair of intronic SNPs from the EPAS1 gene that show strongly elevated allele frequencies in Tibetans compared with Han Chinese. Furthermore, intergenic regions comprise around 70% of the human genome. Many GWAS have established strong associations between intergenic SNPs and diseases [5-7], and many of the results have been replicated in independent datasets. Based on the results from the 1000 Genomes Project [8], among the 1,227 SNPs associated with complex traits identified by GWAS (http://www.genome.gov/gwastudies), under 30% are either annotated as a non-synonymous variant or in substantial LD (r^2^ ≥ 0.5) with a non-synonymous variant.

Exome and targeted sequencing requires capture of DNA fragments that overlap with target regions. Although a captured library is greatly enriched for target regions, a significant fraction of DNA fragments still fall outside target regions. The fraction varies depending on capture efficiency. For example, Yi et al. [4] (using NimbleGen 2.1 M kit) reported having 64.5% of sequenced bases outside target regions and 31.9% more than 500 bp away from target regions; Ng et al. [2] (using Agilent 244 K microarrays for target enrichment) reported over 50% of sequenced bases outside target regions. Our data obtained with Agilent SureSelect v1 capture kit also had nearly 50% of sequenced bases outside target regions. As a result, a significant fraction of intronic and intergenic regions may have been sequenced, which may include promoters, conserved non-coding sequences, untranslated regions, miRNA sites, and other potentially functional regions. It has been a common practice to ignore data outside target regions and focus only on the bases inside target regions [2,4,9-11]. While justified, this approach is inefficient as it overlooks a large amount of data that would otherwise be useful in genetics studies.

In this paper, we systematically evaluate the quality of data outside target regions using our exome sequencing data and a subset of the Pilot 3 data from the 1000 Genomes Project [12]. The Agilent SureSelect v1 capture kit (38 million bases) and Illumina TrueSeq capture kit (62 million bases) used in our exome study are two of the most widely used kits for exome sequencing studies. The 1000 Genomes Project Pilot 3 data focused on the exons of a thousand randomly selected genes as target regions and used Agilent's array enrichment method (2.1 million bases) [8]. For both our data and the data from the 1000 Genomes Project, we will describe coverage, data quality, SNP distribution, and Ti/Tv ratio for the SNPs outside target regions.

## Methods

### Data description

We studied whole exome sequencing data for 28 breast cancer patients recruited to the Shanghai Breast Cancer Study (SBCS). The SBCS is a large, population-based case–control study of women in urban Shanghai, the details of which have been previously described [13]. All patients had very early-onset (22–32 years old) breast cancer or early-onset (38–41 years old) plus first-degree family history of breast cancer. Genomic DNA from buffy coat was extracted using QIAmp DNA kit (Qiagen, Valencia, CA) following the manufacture’s protocol. Approval of the study was granted by the relevant institutional review boards in both China and the United States. All of our samples have been genotyped using the Affymetrix 6.0 array in a previous genome wide association study [13].

We studied 22 exomes captured using the Agilent SureSelect kit and 6 exomes captured using the Illumina TrueSeq kit. Our Agilent SureSelect exome data were 72-base paired-end reads generated from Illumina GA IIx machines. Each sample was run on a single lane of a flowcell. DNA enrichment was done using Agilent SureSelect Human All Exon kit v1 which was designed to target 165,637 genomic regions (37.8 million bases; 71.6% inside exons; average length 228 bp). Our Illumina TrueSeq exome data were 100-base paired-end reads. The six samples were barcoded and sequenced on Illumina HiSeq2000 (five samples per lane). The TrueSeq capture kit targets 201,071 regions (62.1 million bases; 49.3% inside exons; average length 309 bp). The consensus coding sequences database (CCDS) [14] has 27.8 million bases, 98.3% of which are covered by the SureSelect target regions and 96.5% by the TrueSeq target regions. In summary, both kits targeted more than just exon regions but they also did not have 100% coverage of known exons.

We shifted the Illumina base quality scores (Phred + 64) to the Sanger scale (Phred + 33) [15] and performed initial alignment to the NCBI human reference genome (version 36 for SureSelect data and 37 for TrueSeq data) using the program Burrows-Wheeler Aligner (BWA) [16]. We then marked duplicates with Picard [17] and carried out regional realignment and quality score recalibration using Genome Analysis Toolkit (GATK) [18]. For variant calling, we only used reads with a mapping quality score (MAPQ) ≥20 (i.e., ≤1% probability of being wrong) and bases with base quality score (BQ) ≥20. We used GATK's Unified Genotyper to call SNPs simultaneously on all SureSelect samples and then on all TrueSeq samples.

Our SNP filtering criteria were determined through the following steps: 1) Plot Ti/Tv ratio versus genotype quality score (GQ), depth, allelic balance, BQ, and MAPQ for all SNPs and separately for SNPs in dbSNP and novel SNPs. 2) Draw the above plots only for SNPs inside target regions. 3) Calculate overall and heterozygote consistency rates with GWAS data for SNPs overlapping both platforms, filtered by GQ, depth, BQ, and MAPQ. 4) Identify the best filtering criteria that resulted in both high genotype consistency rates and Ti/Tv ratios close to expected values (see next section for details). We found that the two most effective factors affecting sequencing data genotype quality were GQ and depth, and used both GQ ≥ 20 and depth ≥ 5 as genotype filters throughout the study unless otherwise specified.

In addition, we selected 6 subjects from the Pilot 3 study of the 1000 Genomes Project, which designed a capture assay for the exons of 1000 genes (8,496 target regions, 1.4 million bases, average length 169 bp) [8]. Currently, only SNP calls for positions inside the target regions were reported. We selected subjects that were also in the HapMap II so that we could evaluate SNP call quality of sequencing data by comparing them with HapMap genotypes. We focused on Caucasian subjects as a complement to our Asian samples. Two sequencing platforms, Illumina and 454 Life Sciences, were used for those subjects. We focused on the Illumina platform because it used shorter DNA fragments, which tend to overlap more with the typically short target regions. We selected subjects with paired-end data and no flags of “failing” or “withdrawn.” Among those who qualified the above criteria, we selected two subjects from each sequencing center with the highest number of reads: NA12043 and NA12144 from the Sanger Center (SC), which used Nimblegen 385-K array hybridization method; NA12249 and NA12716 from the Washington University Genome Sequencing Center (WUGSC), which used a biotinylated capture library generated by PCR in the presence of biotinylated CTP from a pool of 190 bp synthesized oligos; and NA12154 and NA12005 from the Broad Institute (BI), which used RNA baits transcribed in the presence of biotinylated UTP from primers cleaved from an Agilent microarray. Subject NA12005 had the third highest number of reads from BI; the subject with the second highest number of reads, NA12892, had a very high fraction of non-aligned reads (~10% for one of its two FASTQ files in contrast to ~2% for the other subjects). We downloaded the FASTQ files that were used in the Pilot 3 study and performed the same data processing procedure as that for our samples. For estimation of genotype consistency rate, we downloaded the HapMap II genotype data for these subjects.

### Data quality measurement

One measurement of quality for sequence-based SNP calls is whether they can be validated using an alternative genotyping platform. We thus calculated genotype consistency rate between sequence-based and Affymetrix chip-based SNP calls [13] for SNPs overlapping the two platforms for our subjects, and between sequence-based SNP calls and HapMap genotypes for the subjects from the 1000 Genomes Project. We calculated two types of consistency rate: overall consistency and heterozygous SNP consistency, which probably is more informative on the true error rate. Heterozygote consistency rate was computed as the number of heterozygous genotypes consistent between SNP chip and sequencing divided by the number of heterozygous genotypes on SNP chip that had sequence-based calls with GQ ≥ 20. Furthermore, we calculated Ti/Tv ratio as another measure of data quality. The Ti/Tv ratio is around 3.0 for SNPs inside exons and about 2.0 elsewhere [19]; it also differs between synonymous and non-synonymous SNPs [20]. Since the target regions of exome capture kits often cover more than just exons, the Ti/Tv ratio for SNPs inside target regions is between 2.0 and 3.0 with the value depending on the fraction of exons inside target regions. We also compared the Ti/Tv ratios between novel SNPs and the SNPs reported in dbSNP.

For clarity of presentation, we classified bases into three categories: inside a target region (denoted "inside TR"), outside target regions but within 200 bp from the nearest target region (denoted "outside ≤200 bp"), and outside target regions with >200 bp distance from the nearest target region (denoted "outside >200 bp"). The choice of 200 bp was because the insert sizes of the data we analyzed were mostly between 150 and 200; we also used 100 bp as a threshold and similar patterns of results were observed (data not shown). Reads were classified similarly: a read was "inside TR" if at least half of its bases were inside a target region; similarly, a read was "outside ≤ 200 bp" if at least half the bases were within 200 bp from the nearest target region. The length of a read was determined after applying soft clips according to its CIGAR string information in the BAM file [21]. We studied SNP quality and distribution in the three categories defined above. Furthermore, we annotated SNPs "outside > 200 bp" using the functional variant annotation tool ANNOVAR [22].

## Results

### Quality of SNP chip genotypes

The 22 breast cancer patients sequenced with the Agilent SureSelect capture kit and the 6 samples sequenced with the Illumina TrueSeq capture kit were part of 2776 patients that were genotyped using the Affymetrix SNP 6.0 array in a genome-wide association study; detailed genotyping methods and stringent QC criteria were described in Zheng et al. [13]. The original scan included three quality control samples in each 96-well plate, and the SNP calls showed a very high concordance rate (mean 99.9%; median 100%) for the quality control samples. In addition, 742 SNPs were genotyped using alternative genotyping platforms for a subset of subjects, and they also had a high concordance rate with genotypes obtained from the SNP chip (mean 99.1%; median 99.8%). The SNP chip call rate for the 28 samples investigated here ranged from 97.83% to 99.99%.

### Coverage and distribution of sequence data

Additional file 1: Table S1 contains detailed summaries for the samples we studied. For the 22 samples sequenced with the Agilent SureSelect capture kit, we obtained an average of 68.9 (range 44.6-78.2) million reads per subject, with 45x median depth for the SureSelect target regions. On average, 91.4% (88.4-93.8%) of the reads were aligned to the human reference genome, and 94.2% (92.6-95.5%) had insert size ≤ 500 among the aligned reads. The six samples sequenced with the Illumina TrueSeq capture kit had an average of 93.8 (range 91.3-98.0) million reads and achieved 48x median depth for the TrueSeq target regions. On average, 96.2% (92.4-99.1%) of the aligned reads had insert size ≤ 500. The six samples sequenced by the 1000 Genomes Project had an average of 67.9 (range 47.5-83.7) million reads and achieved 59x median depth for their target regions. On average, 98.6% (98.5-98.8%) of the aligned reads had insert size ≤ 500.

For all three data sets, a significant amount of the reads were more than 200 bases away from target regions (Table 1; details in Additional file 1: Table S2). Among the aligned reads with MAPQ ≥20, our SureSelect data had an average of 56.1% reads (range 53.6-58.8%) "inside TR", 10.4% (9.4-13.4%) "outside ≤200 bp", and 33.5% (30.4-36.0%) "outside >200 bp". Our TrueSeq data had an average of 46.3% reads (range 45.7-46.7%) inside target regions, 21.7% (21.4-21.9%) "outside ≤ 200 bp", and 32.0% (31.5-32.9%) "outside >200 bp". The six samples from the 1000 Genomes Project had an even higher fraction of reads outside target regions: 28.7% (range 26.8-29.6%) "inside TR", 18.6% (8.9-24.9%) "outside ≤ 200 bp", and 52.7% (43.0-64.3%) "outside >200 bp".

**Table 1:** Distribution of reads aligned to the human reference genome: average (standard deviation) in millions, and percentage

	**Overall**	**Inside TR**		**Outside ≤ 200 bp**		**Outside >200 bp**	
SureSelect (n = 22)	# Reads	# Reads	%	# Reads	%	# Reads	%
Before filter	63.4 (9.6)	34.3 (4.9)	54.1	6.4 (1.2)	10.1	22.7 (3.9)	35.8
After filter	57.2 (8.6)	32.0 (4.5)	56.1	6.0 (1.1)	10.4	19.2 (3.3)	33.5
TrueSeq (n = 6)							
Before filter	86.2 (2.5)	37.0 (0.7)	42.9	17.1 (0.3)	19.8	32.1 (1.3)	37.2
After filter	75.1 (2.1)	34.8 (0.7)	46.3	16.3 (0.3)	21.7	24.0 (1.1)	32.0
1000 G (n = 6)							
Before filter	33.9 (8.1)	7.6 (1.2)	22.3	4.7 (1.7)	14.0	21.6 (8.4)	63.7
After filter	25.6 (3.8)	7.3 (1.1)	28.7	4.7 (1.7)	18.6	13.6 (3.6)	52.7

The filter was MAPQ ≥ 20.

As expected, the depth of coverage was the highest for "inside TR" and lowest for "outside >200 bp". For bases around the boundaries of target regions, the average depth follows a clear decreasing pattern as the position moves away from target regions (Figure 1). This is true for all three data sets we analyzed. However, when we focused on reads with MAPQ ≥ 20, the distributions of MAPQ and BQ scores were similar across all three categories of regions we defined (Figure 2). This suggests that after filtering most data outside target regions were as good as those inside target regions. The zigzag pattern in Figure 2 panels (b), (e), and (h) was an artifact of the MAPQ computation algorithm in BWA, which was used to align all the data investigated here.

In our SureSelect data, on average, 97.0% (range 96.5-97.2%) of the sites "inside TR" and 43.8% (38.0-47.7%) of the sites "outside ≤ 200 bp" were covered with at least one read. Of the nearly 3 billion sites "outside >200 bp", an average of 25.5% (14.1-26.1%) were covered with at least one read. Among the sites that had ≥5x depth of coverage, a significant portion still fell outside target regions (Figure 3a): 25.7 million (28.9%) were outside ≤200 bp and 28.2 million (31.7%) were outside >200 bp. The results were similar for our TrueSeq data and the data from the 1000 Genomes Project (Figure 3b,c).

We also examined the GC content of the sequence data we collected. For our SureSelect target regions, the GC content for "inside TR" was 50.6% and dropped to 42.0% for the "outside ≤ 200 bp" regions. For bases "outside > 200 bp" with depth of coverage ≥ 10, the GC content was around 46% (Table 2). Similar patterns were observed for the data sequenced with the Illumina TrueSeq capture kit and by the 1000 Genomes Project (Table 2). The higher GC content inside target regions is a reflection of the known higher GC content of coding regions.

**Table 2:** GC content (base pairs in millions)

		**Inisde TR Outside ≤ 200 bp**		**Outside > 200 bp**		
				**Depth ≥ 30**	**Depth ≥ 20**	**Depth ≥ 10**
SureSelect^a^	total BP	37.8	61.0	2.4	3.4	5.3
(n = 22)	GC	19.1	25.6	1.1	1.6	2.5
	GC%	50.6%	42.0%	45.6%	45.9%	46.4%
TrueSeq^b^	total BP	62.1	76.9	11.3	13.7	18.5
(n = 6)	GC	30.4	32.5	5.1	6.1	8.4
	GC%	49.0%	42.30%	44.9%	44.8%	45.4%
1000 G^c^	total BP	1.4	3.3	0.5	1.3	2.5
(n = 6)	GC	0.7	1.4	0.2	0.6	1.1
	GC%	51.6%	41.6%	46.2%	45.4%	45.2%

^a^ Based on hg18 and Agilent SureSelect v1.

^b^ Based on hg19 and Illumina TrueSeq.

^c^ Based on hg18 and NimbleGen 1.4 M cap kit.

### SNPs inside and outside target regions

We used GQ ≥ 20 and depth ≥ 5 as the thresholds for SNP filtering. For our SureSelect data, we identified 65,231 SNPs inside the target regions, with Ti/Tv ratio 2.81 and heterozygote consistency rate 99.2%. In addition, a total of 84,049 high quality SNPs were identified outside the target regions (Figure 4a,b,c). For "outside ≤ 200 bp" regions, at depth ≥ 5, we observed an average of 44,854 SNPs per subject, with Ti/Tv ratio 2.26; 77.1% of the SNPs were in dbSNP131. The overall consistency rate with array-based SNP calls was 99.5% on average (heterozygote consistency rate 98.9%). For "outside >200 bp" regions, at depth ≥ 5, we observed on average 39,195 SNPs per subject (Ti/Tv ratio 1.83; 68.3% in dbSNP131; overall consistency rate 99.2%; heterozygote consistency rate 98.9%). For "outside >200 bp" regions, the Ti/Tv ratio increased as depth increased; at depth ≥ 20, we observed 15,539 SNPs with Ti/Tv ratio 2.11 and >99% consistency with array-based SNP calls. The detailed SNP numbers and Ti/Tv ratio can be found in Additional file 1: Table S3–S8. The distribution of coverage for SNPs identified outside capture regions can be seen in Table 3.

**Table 3:** Distributions of depth for SNPs outside capture regions that had GQ ≥ 20 and depth ≥ 5

**Depth**	**SureSelect**		**TrueSeq**		**1000 G**	
	**≤ 200 bp**	**> 200 bp**	**≤ 200 bp**	**> 200 bp**	**≤ 200 bp**	**> 200 bp**
5	1156	3463	1225	3963	38	194
6	1000	1681	1278	2950	46	142
7	4036	3630	3200	4702	135	290
8	3483	2570	4005	4582	123	246
9	3040	2020	4222	3995	103	199
10	2647	1658	4231	3447	98	174
11	2349	1420	4261	2972	95	160
12	2099	1251	4223	2628	85	142
13	1866	1096	4188	2366	91	122
14	1694	995	4110	2136	84	115
15	1535	891	4072	1906	84	105
16	1376	823	3949	1749	71	101
17	1270	766	3887	1649	67	91
18	1159	721	3743	1490	67	84
19	1072	672	3654	1421	64	83
≥ 20	15074	15539	63620	62350	1762	1884

The numbers are average numbers of SNPs over the samples from the same capture assay.

For the six samples sequenced with the Illumina TrueSeq capture kit, we identified 95,818 SNPs inside target regions (Ti/Tv ratio 2.30; heterozygote consistency rate 99.6%) and a similar pattern of high quality SNPs outside the target regions (Figure 4d,e,f). For outside ≤ 200 bp regions, at depth ≥ 5, we observed an average of 117,866 SNPs per subject (Ti/Tv ratio 2.01; 87.4% in dbSNP132; overall consistency rate 99.6%; heterozygote consistency rate 99.6%). For outside >200 bp regions, at depth ≥ 5, we observed an average of 104,305 SNPs per subject (Ti/Tv ratio 1.89; 77.3% in dbSNP132; overall consistency rate 99.2%; heterozygote consistency rate 99.1%).

For the six samples sequenced by the 1000 Genomes Project, we identified 1,548 SNPs inside their target regions (Ti/Tv ratio 3.23; heterozygote consistency rate 99.3%) and many high quality SNPs outside the target regions (Figure 4g,h,i). For outside ≤ 200 bp regions, at depth ≥ 5, we observed an average of 3,011 SNPs per subject (Ti/Tv ratio 2.23; 92.5% in dbSNP131; overall consistency rate 98.6%; heterozygote consistency rate 99.6%). For outside >200 bp, at depth ≥ 5, we observed an average of 4,128 SNPs per subject (Ti/Tv ratio 1.80; 85.3% in dbSNP131; overall consistency rate 97.2%; heterozygote consistency rate 98.7%). At depth ≥ 20, we observed 1,883 SNPs with Ti/Tv ratio 1.96 and >98% consistency with HapMap data.

We used ANNOVAR to annotate the SNPs identified more than 200 bp away from target regions. The characteristics of those SNPs are summarized in Table 4. For sites with average depth ≥ 5 in our SureSelect data, we observed 6,194 SNPs in introns, 35 within 2 bp of a splicing junction, 4,873 in noncoding RNAs (ncRNAs), and 33,569 intergenic. We were also able to observe many exonic SNPs that were outside the target regions of the SureSelect or TrueSeq capture kit used for generating our data, including 981 non-synonymous SNPs and 25 stop-gain and 4 stop-loss mutations. These potentially functional SNPs could be missed if the investigator would only look at results inside target regions. We also summarized the results for sites with average depth ≥10, and repeated this procedure for our TrueSeq data and the data generated by the 1000 Genomes Project (Table 4).

**Table 4:** SNPs > 200 bp outside target regions

**Samples**	**Average depth**	**Intronic**	**Splicing^a^**	**ncRNA^b^**	**Intergenic**	**Exonic**		
						**Non-synonymous**	**Stopgain**	**Stoploss**
SureSelect	≥ 5	6194	35	4873	33569	981	25	4
(n = 22)	≥ 10	4297	34	3790	21713	792	17	3
TrueSeq	≥ 5	29372	8	7537	74431	211	4	0
(n = 6)	≥ 10	16252	7	10153	60889	199	4	0
1000 G	≥ 5	2619	11	309	1951	307	6	1
(n = 6)	≥ 10	1352	8	168	1183	213	5	1

^a^ Variant is within 2-bp of a splicing junction.

^b^ Variant overlaps a transcript without coding annotation in the gene definition.

## Discussion

Our samples were blood samples from breast cancer patients. Though they were not a random sample, we expect this to have little impact on the generalizability of the phenomena we observed. We did not evaluate any data generated by sequencers from 454 Life Sciences or Applied Biosystems. However, we expect similar results for these platforms because the SureSelect whole exome capture kit we used was designed to be compatible with all three major platforms of sequencing technology.

We examined if SNP quality could be influenced by artifacts. One artifact of exome capturing is strand imbalance; that is, the distribution of forward and reverse strands can be heavily uneven at many positions, especially those close to the boundaries of target regions. This phenomenon exists for positions both inside and outside target regions, although it is more extreme outside target regions. In extreme situations, all reads can be on the same strand (examples in Additional file 1: Table S9). Using our Agilent SureSelect data, we selected all positions where the depth was ≥5, all reads were on one strand, and GWAS genotypes were available; 46,069 positions only had forward reads, and 43,145 positions only had reverse reads. We computed genotype consistency rates with GWAS data for those positions. Both overall and heterozygous consistency rates were above 98.5%. Thus, the strand imbalance had little effect on genotype quality.

Another artifact we observed with exome sequencing is that some regions far away from target regions had abnormally high coverage, with depth in the hundreds. We suspected that this high coverage might be a result of strong homology between intergenic regions and exome regions, causing nonspecific binding during the capture process. Using our Agilent SureSelect data we picked 10 regions with very high depth (Additional file10: Table S10) and examined them against the pseudo gene lists from Yale and UCSC (http://www.pseugenes.com). Only 2 regions overlapped with pseudo genes. We also BLASTed [17] these 10 intervals against the SureSelect target regions and found that most of these intervals had strong homology with sequences inside the target regions (Additional file 1: Table S10). Our analysis results on GC content also suggest that many of the high depth regions "outside > 200 bp" have strong homology with coding regions and thus a higher GC content than introns. Further study is needed to understand the full extent of sequence homology in the human genome.

In addition to SNPs, we found many indels outside target regions that had high quality scores. For our data sequenced with the Agilent SureSelect capture kit, filtered with QUAL Score (reported by GATK's Unified Genotyper) ≥ 1000, 2,383 indels were detected by GATK's Unified Genotyper inside the target regions, 5,344 were outside ≤ 200 bp, and 4,158 were outside > 200 bp. The numbers were 7,211, 9,456, and 7,426 for the six TrueSeq samples, and 10, 273, and 207 for the six samples sequenced by the 1000 Genomes Project. Systematic validation of these indel calls would take a significant amount of effort and is beyond the scope of this paper.

The amount of information outside target regions depends on capture efficiency, which varies across different capturing technologies. With Agilent’s SureSelect v1 capturing kit, only 55% of the reads in our data were aligned within target regions; it was about 50% for Illumina’s TrueSeq capture kit. In general, older capture kits were less efficient compared to the newer kits. But even the most current capture kit today can only claim to have around 80% capture efficiency [23], while the real capture efficiency is probably lower in practice. We have shown little quality deterioration outside target regions after filtering. Thus for exome and targeted sequencing data, instead of inefficiently limiting ourselves to variants inside target regions, we should also analyze data in any regions with high-quality SNP calls.

Furthermore, exome sequencing has also been used with non-human subjects. Regardless of the organism being sequenced, our findings about data outside target regions should apply. By including SNPs outside target regions, the genomic coverage is effectively improved and the chance of identifying quantitative trait loci is increased.

## Conclusion

We studied the quality of SNP calls for positions outside target regions using our whole exome sequencing data and the 1000 Genomes Project Pilot 3 data. These datasets were generated using five different capture kits and three different sets of target regions. The sequencing was also performed at five different facilities. Despite these differences, we observed reliable genotypes for many SNPs outside target regions, some far outside target regions, in all datasets. By analyzing all available sequencing data and applying stringent filtering criteria, we more than doubled the number of high-quality SNP calls in comparison to what we would have if we had just focused on target regions. Given the amount of significant discovery researchers have made in the noncoding regions, these extra SNPs residing outside target regions should not be ignored in data analysis, especially given the fact that sequencing is still relatively expensive.

## Competing interests

The authors declare that they have no competing interests.

## Authors’ contribution

C Li and J Long designed the study. Y Guo performed most of the analyses, with substantial help from J He and CI Li. Y Guo and C Li wrote the paper. J Long, W Zheng, XO Shu, and Q Cai provided valuable suggestions and commented on the manuscript. Q Cai was in charge of the sample preparation. All authors contributed to the manuscript.

## Supplementary Material

Additional file 1 **Table S1.** Alignment Summary Table (read in millions). **Table S2.** Agilent SureSelect v1 (N=22, MAPQ>20). **Table S3.** SNP information for 22 SureSelect samples. **Table S4.** SNP information for 22 SureSelect samples (only considering dbSNP overlap). **Table S5.** SNP information for 6 1000 Genome Project samples. **Table S6.** SNP information for 6 1000 Genome Project samples (only considering dbSNP overlap). **Table S7.** SNP information for 6 TrueSeq samples. **Table S8.** SNP information for 6 TruSeq samples (only considering dbSNP overlap). **Table S9.** Positions with unbalanced read distribution in SureSelect data. **Table S10.** Blast Results for top 10 high depth Outside > 200bp regions in Agilent SureSelect Regions. Click here for file

## References

[B1] Ng SB Targeted capture and massively parallel sequencing of 12 human exomes Nature 2009 461 7261 272 276 10.1038/nature08250 19684571 PMC2844771

[B2] Ng SB Exome sequencing identifies the cause of a mendelian disorder Nat Genet 2010 42 1 30 35 10.1038/ng.499 19915526 PMC2847889

[B3] Rearick D Critical association of ncRNA with introns Nucleic Acids Res 2011 39 6 2357 2366 10.1093/nar/gkq1080 21071396 PMC3064772

[B4] Yi X Sequencing of 50 human exomes reveals adaptation to high altitude Science 2010 329 5987 75 78 10.1126/science.1190371 20595611 PMC3711608

[B5] Hancock DB Genome-wide association study implicates chromosome 9q21.31 as a susceptibility locus for asthma in mexican children PLoS genetics 2009 5 8 e1000623 10.1371/journal.pgen.1000623 19714205 PMC2722731

[B6] Wright FA Genome-wide association and linkage identify modifier loci of lung disease severity in cystic fibrosis at 11p13 and 20q13.2 Nat Genet 2011 43 6 539 546 10.1038/ng.838 21602797 PMC3296486

[B7] Einarsdottir E Multiple independent variants in 6q21-22 associated with susceptibility to celiac disease in the Dutch, Finnish and Hungarian populations European journal of human genetics : EJHG 2011 19 6 682 686 10.1038/ejhg.2011.2 21326284 PMC3110053

[B8] Durbin RM A map of human genome variation from population-scale sequencing Nature 2010 467 7319 1061 1073 10.1038/nature09534 20981092 PMC3042601

[B9] Yan XJ Exome sequencing identifies somatic mutations of DNA methyltransferase gene DNMT3A in acute monocytic leukemia Nat Genet 2011 43 4 309 315 10.1038/ng.788 21399634

[B10] Nikolaev SI Exome sequencing identifies recurrent somatic MAP2K1 and MAP2K2 mutations in melanoma Nat Genet 2012 44 2 133 139 22197931 10.1038/ng.1026

[B11] Vissers LE Whole-exome sequencing detects somatic mutations of IDH1 in metaphyseal chondromatosis with D-2-hydroxyglutaric aciduria (MC-HGA) American journal of medical genetics. Part A 2011 155A 11 2609 2616 22025298 10.1002/ajmg.a.34325

[B12] DePristo MA A framework for variation discovery and genotyping using next-generation DNA sequencing data Nat Genet 2011 43 5 491 498 10.1038/ng.806 21478889 PMC3083463

[B13] Zheng W Genome-wide association study identifies a new breast cancer susceptibility locus at 6q25.1 Nat Genet 2009 41 3 324 328 10.1038/ng.318 19219042 PMC2754845

[B14] NCBI CCDS Available from: [http://www.ncbi.nlm.nih.gov/projects/CCDS/CcdsBrowse.cgi]

[B15] Cock PJ The Sanger FASTQ file format for sequences with quality scores, and the Solexa/Illumina FASTQ variants Nucleic Acids Res 2010 38 6 1767 1771 10.1093/nar/gkp1137 20015970 PMC2847217

[B16] Li H Durbin R Fast and accurate short read alignment with Burrows-Wheeler transform Bioinformatics 2009 25 14 1754 1760 10.1093/bioinformatics/btp324 19451168 PMC2705234

[B17] NCBI BLAST Available from: [http://blast.ncbi.nlm.nih.gov/Blast.cgi]

[B18] McKenna A The Genome Analysis Toolkit: a MapReduce framework for analyzing next-generation DNA sequencing data Genome Res 2010 20 9 1297 1303 10.1101/gr.107524.110 20644199 PMC2928508

[B19] Bainbridge MN Targeted enrichment beyond the consensus coding DNA sequence exome reveals exons with higher variant densities Genome Biol 2011 12 7 R68 10.1186/gb-2011-12-7-r68 21787409 PMC3218830

[B20] Yang Z Nielsen R Synonymous and nonsynonymous rate variation in nuclear genes of mammals J Mol Evol 1998 46 4 409 418 10.1007/PL00006320 9541535

[B21] Li H The Sequence Alignment/Map format and SAMtools Bioinformatics 2009 25 16 2078 2079 10.1093/bioinformatics/btp352 19505943 PMC2723002

[B22] Wang K Li M Hakonarson H ANNOVAR: functional annotation of genetic variants from high-throughput sequencing data Nucleic Acids Res 2010 38 16 e164 10.1093/nar/gkq603 20601685 PMC2938201

[B23] NimbleGen Nimblegen Seqcap Available from: [http://www.nimblegen.com/products/seqcap/]

